# Factors Associated with Perceived Continuation of Females' Genital Mutilation among Women in Ethiopia

**Published:** 2010-03

**Authors:** Zenebe Fikrie

**Affiliations:** 1 Department of Statistics, College of Computing and Sciences, Jimma University

**Keywords:** Female genital Mutilation, Predictor, Ethiopia

## Abstract

**Background:**

Females genital mutilation is one of the harmful traditional practices affecting the health of women and children. It has a long-term physiological, sexual and psychological effect on women. Females' genital mutilation still remains to be a serious problem for large proportion of women in most sub-Saharan Africa countries including Ethiopia. The objective of the study was to identify the main factors contributing to the support for the continuation of female genital mutilations in Ethiopia.

**Methods:**

This study was conducted based on secondary data obtained from the Ethiopian Demographic and Health Survey 2005. A two-stage stratified cluster sampling design was applied for selecting the sampling units. Both descriptive and binary logistic regression analyses were used to analyse the data using SPSS for Windows version 16.

**Results:**

The results on both descriptive and logistic regression model revealed that predictor variables like education, religion, residence, knowledge on ways of HIV transmission and region play significant role in determining the dependent variable. As a result, all predictor variables were strongly associated with the dependent variable. Regarding the fit of the model, support for the continuation of FGM, decreased with increase in education status. Furthermore, Muslim among other religions, and Somali and Afar among other regions, were more likely to support for the continuation of FGM.

**Conclusion:**

There was low awareness with less education status, Somali and Afar regions, rural residence, and Muslim religion were predictors of continuation of females' genital mutilation.

## Introduction

Among the harmful traditional practices, females' genital mutilation (FGM) was the focus of this study. FGM is described as all procedures involving partial or total removal of external female genitalia or other injury to the female genital organs for cultural or non-therapeutic reasons (1). This practice has long-term physiological, sexual and psychological effects on women, among which the immediate consequences are pains, bleeding, haemorrhage, shock, acute infection, and transmission of HIV/AIDS due to unclean conditions often associated with the procedure (2). Similarly, the long term consequences include menstrual disorder, painful sexual intercourse, loss of sexual appetite (frigidity), fistula, psychological consequences, etc (2). Between 100 and 140 million girls and women in the world are estimated to be circumcised, and 3 million girls are estimated to be at the risk of being circumcised every year (3). This statement depicts that FGM still persists despite the fact that several measures have been taken by local communities, governments, and national as well as international organizations to eliminate it.

Different justifications for the persistence of FGM are given in different societies. In some of them, girls pass through such procedure because it is accepted as an important part of their cultural identity; while for others, it is just for fear of stigmatization and rejection by their community. There are also other justifications such as religion and girls' marriageability (1). These justifications indicate that FGM is a manifestation of gender inequality that is deeply entrenched in social, economic and cultural structures. FGM is most prevalent in northeastern Africa countries where the prevalence varies from 97% in Egypt to 80% in Ethiopia (4). Several measures have been taken to bring about awareness on the harmful consequences of FGM over the past decade at international, regional, and national levels. In spite of these efforts, prevalence of FGM in many areas remains high. And this makes the study relevant and timely that plays important role in identifying the main factors contributing to support for the continuation of FGM in Ethiopia.

## Methods and Materials

This study was based on secondary data, where the source of the data is the Central Statistical Agency (CSA). This data set was generated by the survey called Ethiopian Demographic and Health Survey (EDHS) carried out in 2005.

The study covered the entire country in which both rural and urban areas are considered. All the nine regions and two city administrations, namely Tigray, Afar, Amhara, Oromiya, Somali, Benshangul-Gumuz, Southern Nations, Nationalities and Peoples (SNNP), Gambela, Harari, Addis Ababa and Dire Dawa were included in the survey which was conducted from April 27 through August 30, 2005.

A two-stage stratified clustered sampling design was used in EDHS 2005. In the survey, a representative sample of approximately 14,500 households from 540 clusters was selected. In the first stage, 540 clusters (145 urban and 345 rural) were selected from the list of Enumeration Areas (Primary Sampling Units) from the 1994 Population and Housing Census sample frame. As part of the second stage selection, a complete household listing was carried out in each selected cluster. The listing operation yields between 24 and 32 households from each cluster, where the selection of these households (Secondary Sampling Units) was done systematically and made ready for the survey.

The 2005 EDHS sample was designed to provide estimates for the health and demographic variables for the domains; Ethiopia as whole, urban and rural areas of Ethiopia (each as a separate domain) and 11 geographic areas (9 regions and 2 city administrations).

The survey employed three types of questionnaires namely household, women's and male's questionnaires (household questionnaire, women's and male's questionnaires). The women's questionnaire, which involves harmful traditional practices, was the focus of this study. Information had been gathered from 14,070 women of age 15 – 49 years using the structured women's questionnaire during the survey.

The actual data collection was conducted at the field level by 180 trained interviewers supervised by 30 field supervisors in which the entire field operation was coordinated by the CSA.

The dependent variable was a request raised for all women who participated in the survey as to whether they support for the continuation of FGM practice; expected binary responses was “yes” or “no”. Some of the corresponding independent variables included were educational status, religion, residence and region. Data were analyzed using SPSS for Windows version 16. Descriptive statistics was also used as the method of data analysis in addition to fitting the logistic regression model. The data that corresponds to the variables considered by this study were extracted from the set of the DHS data particularly compiled from the women's questionnaire.

As the author was member of the CSA during the study time, permission to use the data was obtained from the Deputy Manager after explaining the purpose of the analyses.

## Results

The support for the continuation of females' genital mutilation decreased from 42.8% (no education) to 2.0% (higher education). It was also observed that the support for the continuation of the practice ranged from 76.0% (Somali) and 69.0% (Afar) to 13.3% (Dire Dawa) and 5.9% (Addis Ababa), respectively. The result for the other regions remained in between. Similarly, the support for the continuation of FGM with respect to religion decreased from 45.8% among Muslims to 20.1% among Protestants. All socio-demographic variables were associated with continuation of FGM (p<0.05) (Table 1).

**Table 1:** Socio-demographic characters of women aged 15 – 49 by their support for the continuation of FGM, Ethiopia, 2005.

Predictor variable	Category of variables		Should FGM be continued?			
		No		Yes		
		Number	%	Number	%	
Education status	No education	4228	57.2	3168	42.8	0.000
	Primary	2201	82.2	475	17.8	
	Secondary	2164	95.7	97	4.3	
	Higher educ.	349	98.0	7	2.0	
Religion	Orthodox	4923	79.2	1294	20.8	0.000
	Catholic	95	75.4	31	24.6	
	Protestant	1440	79.9	363	20.1	
	Muslim	2382	54.2	2013	45.8	
	Traditional	99	68.3	46	31.7	
Residence	Rural	5049	60.3	3319	39.7	0.000
	Urban	3893	90.1	428	9.9	
Partner's education status	No education	2807	53.7	2425	46.3	0.000
	Primary	1489	72.6	561	27.4	
	Secondary	1242	87.1	184	12.9	
	Higher	345	96.6	12	3.4	
Region	Tigray	798	77.6	231	22.4	0.000
	Afar	240	31.0	535	69.0	
	Amhara	1059	61.2	672	38.8	
	Oromiya	1529	70.3	646	29.7	
	Somali	158	24.0	499	76.0	
	Benshangul-G	437	65.6	229	34.4	
	SNNP	1349	74.7	458	25.3	
	Gambela	271	79.7	69	20.3	
	Harari	650	77.2	192	22.8	
	Addis Ababa	1752	94.1	109	5.9	
	Dire Dawa	699	86.7	107	13.3	

The logistic regression analysis was consistent with the above finding. According to the model output with respect to education status, it was observed that those respondents whose education at primary level were 0.65 times less likely to support for the continuation of FGM compared to those with no education level [OR = 0.65, 95% C.I: (0.55, 0.77)]. Similarly, those with higher education level were 0.14 times less likely to support for the continuation of the practice than those with no education [OR = 0.14, 95% C.I: (0.03, 0.62)]. Muslims were 1.34 times more likely to support for the continuation of the practice than Orthodox [OR = 1.34, 95% C.I: (1.19, 1.53)]. Furthermore, the odds ratio for both Catholic and Protestants, respectively, were 0.52 and 0.71 times less likely to support for the continuation of the practice as compared to Orthodox [OR = 0.52, 95%C.I: (0.26, 1.01); OR = 0.71, 95% C.I: (0.59, 0.85)]. The result further showed that those who have knowledge of avoiding HIV/AIDS was 0.60 times less likely to support for the continuation of FGM compared to their counter parts [OR = 0.60, 95% C.I: (0.54, 0.68)] (Table 2).

**Table 2:** Logistic regression analysis used to identify contributing factors to support for the continuation of FGM, Ethiopia, 2005.

Predictor Variables	Coeff (β)	S.E	Wald	d.f	O.R	(95% C.I )	
**Partner's age**			16.15	5			
20–24	0.52	0.51	1.05	1	1.68	0.624	4.51
25–29	0.73	0.50	2.13	1	2.07	0.780	5.48
30–39	0.84	0.49	2.89	1	2.31	0.88	6.09
40–49	0.66	0.49	1.80	1	1.94	0.74	5.13
50 & above	0.58	0.50	1.36	1	1.78	0.67	4.71
**Religion**			58.87	4			
Catholic	−0.66	0.34	3.72	1	0.52	0.26	1.01
Protestant	−0.35	0.09	13.65	1	0.71	0.59	0.85
Muslim	0.30	0.06	20.89	1	1.34	1.18	1.53
Traditional	−0.11	0.23	0.22	1	0.90	0.57	1.41
**Residence**	0.99	0.11	85.80	1	2.69	2.18	3.31
**Education status**			51.71	3			
Primary education	−0.43	0.09	23.76	1	0.65	0.55	0.77
Secondary education	−1.13	0.20	33.53	1	0.32	0.22	0.47
Higher education	−1.95	0.75	6.79	1	0.14	0.03	0.62
**Partner's education**			49.61	3			
Primary education	−0.44	0.07	37.48	1	0.64	0.56	0.74
Secondary education	−0.55	0.12	19.87	1	0.58	0.45	0.74
Higher education	−1.17	0.40	8.53	1	0.31	0.14	0.68
**Knowledge about HIV**	−0.50	0.06	71.20	1	0.60	0.54	0.68
**Circumcision status**	1.32	0.11	142.80	1	3.76	3.03	4.67
**Constant**	−2.75	0.52	27.97	1	0.06		

It can be observed from the outputs that both results of the two analyses are consistent.

## Discission

This study showed that education modified the attitude towards the continuation of FGM. Both the descriptive and the logistic regression analyses of this study revealed that increase in education status results less support to the continuation of FGM. That is, the more people are educated the more they disregard the continuation of FGM (98% and 57.7% rejection of the continuation of FGM by higher education and by no education levels, respectively), making this result consistent with findings from other study.

Women with higher education are expected to have more access and exposure to media and advocacy messages and possess greater awareness of the humane right implications (4). It is also shown that the ratios of the proportions of women aged 15 – 49 who indicated that FGM should continue varies according to their level of education. It is mentioned that in 10 of 15 African countries, for which data are available, support for the continuation of FGM is higher among women with no education, compared to those with some education (4).

Large proportion of women indicated that they believe religion requires FGM (4). It was also mentioned that in 9 out of the 12 surveyed countries that collected information on religion, greater proportion of Muslim women supported the continuation of FGM (4). Differences in the levels of supporting for the continuation of FGM between Muslim and Christian women was particularly apparent in Eritrea (Muslim - 73%, Christian - 32%), Ethiopia (Muslim - 76%, Christian - 58%), and northern Sudan (Muslim - 79%, Christian - 42%). Similarly, this study showed that Muslim religion (48.8%) compared to orthodox religion (20.8%), was more likely to support for the continuation of FGM. It was also more likely compared to Catholic (24.6%) and Protestant (20.1%) religions with respect to the support for the continuation of FGM. It was also observed from the result of this study that Somali (78.2%) and Afar (72%) regions among other regions were more likely to support for the continuation of FGM.

In conclusion, the study showed that women with low educational level, from Muslim religion, from remote region and from rural residence had less awareness about harmfulness of FGM. Governmental or non-governmental organizations should make intensive programs of informal education, as well as awareness raising trainings through different social groups such as Edir and holydays celebrations with emphasis to Muslim dominant areas and rural residents.

## References

[1] World Health Organization (2008). Eliminating Female Genital Mutilation.

[2] World Health Organization (2008). Female genital mutilation.

[3] UNICEF (2006). Progress in sexual and reproductive health research.

[4] UNICEF (2005). Female Genital Mutilation/Cutting.

[5] National Committee on Traditional Practices of Ethiopia (2003). Old beyond Imaginings.

[6] Central Statistical Agency and ORC Macro (2005). Ethiopian Demographic and Health Survey.

